# Patterns of milking unit kick-off as a proxy for habituation to milking in primiparous cows

**DOI:** 10.3168/jdsc.2023-0384

**Published:** 2023-07-21

**Authors:** D. Kness, T. Grandin, J. Velez, J. Godoy, D. Manríquez, F. Garry, P. Pinedo

**Affiliations:** a Department of Animal Sciences, Colorado State University, Fort Collins, CO 80523; b Aurora Organic Farms, Platteville, CO 80651; c École Nationale de Vétérinaire de Toulouse, CIRAD, Toulouse 31300, France; d Department of Clinical Sciences, Colorado State University, Fort Collins, CO 80523

## Abstract

The onset of lactation and the subsequent habituation to the milking routine is a stressful period, particularly for primiparous (PRI) cows. The objective of this study was to describe the dynamics of milking unit kick-off (KO) behavior in PRI cows during the first 3 mo of lactation, considering multiparous (MUL) cows as a reference for comparison. In addition, the potential associations between KO and milk yield and mastitis presentation were investigated. A total of 869 cows (PRI = 199; MUL = 670) on a dairy farm in northern CO were included in the analysis. Cows calving between August and November 2020 were enrolled from 3 DIM until 90 DIM. Participants were milked 3×/day in a 60-unit rotary parlor and data from each milking session were downloaded from parlor management software. Milking unit kick-off was used as a proxy for habituation to the milking procedure. Kick-off events were reported by the milking system and defined as an abrupt interruption in the milk flow during the milking process. Cow KO events were recorded for each milking session. Subsequently, occurrence of KO was analyzed by grouping the 3 consecutive milking sessions in each day and categorized as yes or no, indicating whether or not an individual kicked at least once in a given day. Data were analyzed by repeated measures ANOVA and logistic regression, including parity category, calving season, occurrence of dystocia, and their potential interactions in the models. Least squares means for daily proportions of KO were calculated considering the number of cows with KO events per day in the PRI and MUL categories. Subsequently, odds ratios for the occurrence of KO in PRI versus MUL were calculated at multiple periods of time. When KO was analyzed by DIM, proportions of KO were greater in PRI than in MUL during the whole monitoring period. In PRI, proportions of KO increased from 0.10/d to 0.20/d between 3 DIM and 15 DIM, to start decreasing around 30 DIM and remaining above MUL up to 90 DIM. On the contrary, in MUL cows, proportions of KO remained close to 0.05/d during the 90-d period. Overall, the odds of KO were greater for PRI versus MUL cows (odds ratio [95% confidence interval] = 2.07 [1.58–2.73]). No differences in milk yield were established among KO categories, while the percentage of cows affected with mastitis was greater in cows grouped in the quartile with more frequent KO events. We concluded that the relationship between days in milk and the proportion of PRI cows displaying KO was not linear, but rather KO increased during the first 2 wk postpartum before decreasing after the first month of lactation. First-parity cows had greater levels of KO than MUL cows, which is most likely associated with the process of habituation to milking during their first lactation.

## Highlights

• Milking unit kick-off has the potential to affect worker and animal welfare, as well as cow performance.
• Occurrence of KO in MUL cows was consistent throughout the monitoring period, but PRI cows experienced periods of greater KO between the second and the fourth week of lactation.
• Compared with MUL, PRI cows had doubled odds of KO during the first 90 days in milk.

## Graphical Abstract

**Figure:**
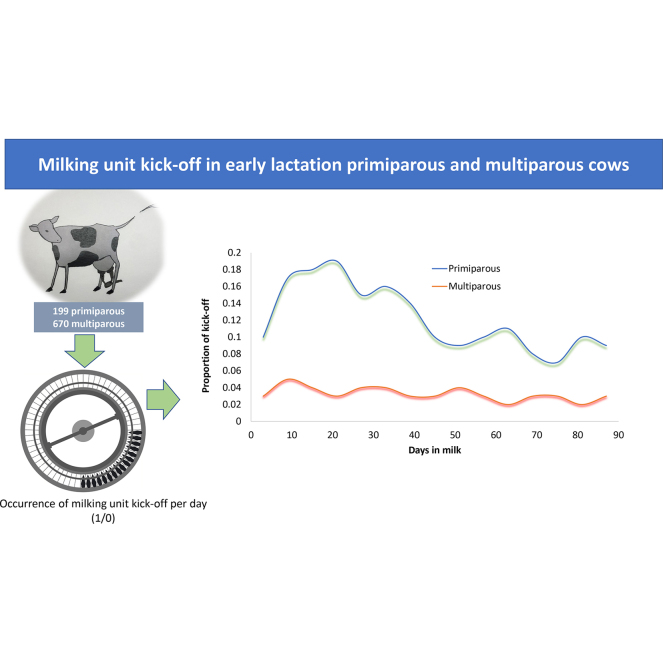
**Summary:** Habituation to the milking routine is a stressful process for primiparous cows, and animal reactions to new settings may represent a risk for both workers and cows. The objective of this study was to analyze the dynamics of milking unit kick-off (KO) behavior in primiparous (PRI) and multiparous (MUL) cows during the first 3 months of lactation. The odds of KO in PRI were higher than those of MUL during the entire monitoring period. The proportions of KO in PRI cows increased during the first week postpartum before decreasing after the first month of lactation, whereas KO in MUL remained stable during the whole monitoring period. Our results indicate that KO behavior in PRI cows persists over the course of the lactation, but the frequency of KO events declines after the first 30 days in milk.

Acute stress related to a negative affective state and stimulation of a fight-or-flight response in dairy cows has well-known, detrimental effects on welfare and productivity and can have an impact on cattle handling and worker safety (Grandin, 1993, 1999). On commercial dairy operations, animal handling-related injuries comprise between 24% and 38% of all reported injuries, with around 20% of those involving moving animals to the parlor and 50% occurring in the milking parlor itself (Grandin et al., 1998; Lindahl et al., 2016; Edwards and Kuhn-Sherlock, 2021). Many behaviors that pose a risk to human handlers, such as kicking and crushing handlers against the pen, are in response to agitation and acute stress and most often occur while the handler is attaching the milking unit or completing other milking-related tasks (Grandin et al., 1998; Douphrate et al., 2013; Edwards and Kuhn-Sherlock, 2021).

The onset of the first lactation and the subsequent period of habituation to the milking routine is a particularly stressful period in a dairy cow's life, with an increase in interaction with human caretakers, new social groups, and a host of novel stimuli during the milking routine. Handling primiparous (**PRI**) heifers during the transition period can also negatively affect human handlers, with increased difficulty of performing milking tasks and risk of cattle-related injuries (Sorge et al., 2014; Edwards and Kuhn-Sherlock, 2021; Phillips et al., 2021). Despite this, there is a gap in research regarding the specific changes in cow behavior over the course of the first lactation, which limits the scope of training that animal caretakers can receive.

Previous studies on heifer habituation to the milking routine indicate that PRI cows tend to be more excitable than multiparous (**MUL**) cows at various stages of the milking process (Szentléleki et al., 2015). The majority of research has primarily focused on shortening the habituation process through the use of pre-lactation exposure to the milking routine or contact with caretakers on early lactation behaviors and heifer habituation (Bremner, 1997; Arnold et al., 2007; Eicher et al., 2007; Kutzer et al., 2015). However, to the authors' knowledge, no studies describe daily changes in stress behaviors during the habituation period or differences in those daily behaviors between PRI and MUL cows during the first months of lactation.

The behavioral responses that dairy cows display to novel stimuli (such as kicking) pose significant risk of injury to the worker, creating a need for realistic expectations regarding the changes in behavior over the habituation process. Because of this, a better understanding of the habituation process is especially relevant to handlers working in the milking parlor, as they come in close contact with the animals on a daily basis.

We hypothesized that milking unit kick-off (**KO**) would be most frequent at the beginning of lactation of PRI cows, declining as the lactation advances. The primary objective of this study was to describe the dynamics of KO behavior in PRI cows during the first 3 mo of lactation and to compare these changes with those of MUL cows over the same period of time. In addition, the potential association between KO and milk yield and mastitis presentation were investigated.

Behavioral and milk production data were collected from an organic-certified dairy farm in northern Colorado using a parlor management software (DelPro Farm Manager software; DeLaval International AB, Tumba, Sweden). A total of 869 Holstein cows (199 PRI and 670 MUL) that calved between August and November 2020 were enrolled in the study at 3 DIM and monitored until 90 DIM. As all the data used in this study were collected from on-farm software, no Colorado State University Institutional Animal Care and Use Committee approval was required. Study cows were milked 3 times per day in a 60-unit rotary parlor (DeLaval International AB). The main variable of interest was milking unit kick-off (categorized as yes or no), monitored with the electronic on-farm milk meters (DeLaval model MM27BC). The meter measures milk yield, milk flow, and milking duration by use of infrared light technology. Kick-off occurrence, detected through these measurements, is reported and stored in the parlor management software, where the records become available for transferring into Excel (Microsoft Corp.) spreadsheets. Milking unit kick-off was identified by the milking system as an abrupt interruption in the milk flow during the milking process and considered in this study as a proxy for habituation to the milking routine.

Data pertaining to individual cows, such as parity, calving date, and calving ease score, were exported from PCDART herd management software (Dairy Records Management Systems). Reports were then generated and downloaded via a remote server once weekly for the duration of the study, which lasted until the last cow enrolled had completed her first 90 DIM.

Data exploration and descriptive analyses for daily average milk yield and daily average milk flow were performed using PROC MEANS and PROC GLM (SAS 9.4; SAS Institute Inc., Cary, NC).

Cow KO events were recorded for each milking session. Subsequently, occurrence of KO was analyzed by grouping the 3 consecutive milking sessions in each day and categorized as yes or no, indicating whether or not an individual kicked at least once in a given day. In addition, numbers of days with a KO event were categorized for each cow into quartiles as **Q1** (<2), **Q2** (≥2 d to ≤ 3 d), **Q3** (>3 d to ≤6 d), and **Q4** (>6 d) (PROC UNIVARIATE) for milk yield and occurrence of mastitis comparisons. Initial univariable models using only parity category (PRI and MUL) as explanatory variable were followed by multivariable models that added calving season, calving difficulty (1 = no assistance; 4 = extreme force), and their potential interactions as covariables. Odds ratios (**OR**) for KO were calculated for PRI and MUL cows using PROC GLIMMIX for the whole study period and by weekly periods.

The logistic equation to investigate the effects of parity can be expressed as presented by de Mutsert et al. (2009):

ln [*p*/(1 − *p*)] = β_0_ + β_1_(parity) + β_2_(COV) + β_3_(parity × COV),

where ln is the natural logarithm; *p* is the proportion of cows registering KO and [*p*/(1 − *p*)] are the odds of this outcome; β_0_ is the model intercept for the study outcome; and β_1_, β_2_, and β_3_ are the regression parameters for parity category, the proposed covariables (COV), and the interaction term parity × COV.

Least squares means for the daily proportions of KO in PRI and MUL cows were calculated using PROC GENMOD. A backward stepwise selection approach was used considering the 2 categories for parity, the covariables (COV), and their interactions in the initial model. Significant predictors were selected at *P*-value < 0.05; interaction terms remained in the models at *P*-value ≤ 0.10.

The study group consisted of 199 PRI and 670 MUL cows. A total of 4 study cows (1 PRI, 3 MUL) left the milking herd before completion of the monitoring period, but data collected from these cows before leaving the herd were still included in the analysis.

Figure 1 provides daily milk yield (kg/d) and average milk flow (kg/min) during the study period for the participant cows separated by parity category. Though not directly related to the main goal of this study, data were consistent with other research on the relationship between parity and milk production, with daily yield and flow rate being higher in MUL cows than PRI (Erb and Martin, 1980; Firk et al., 2002; Lee and Kim, 2006; Ben Meir et al., 2019).

**Figure 1:**
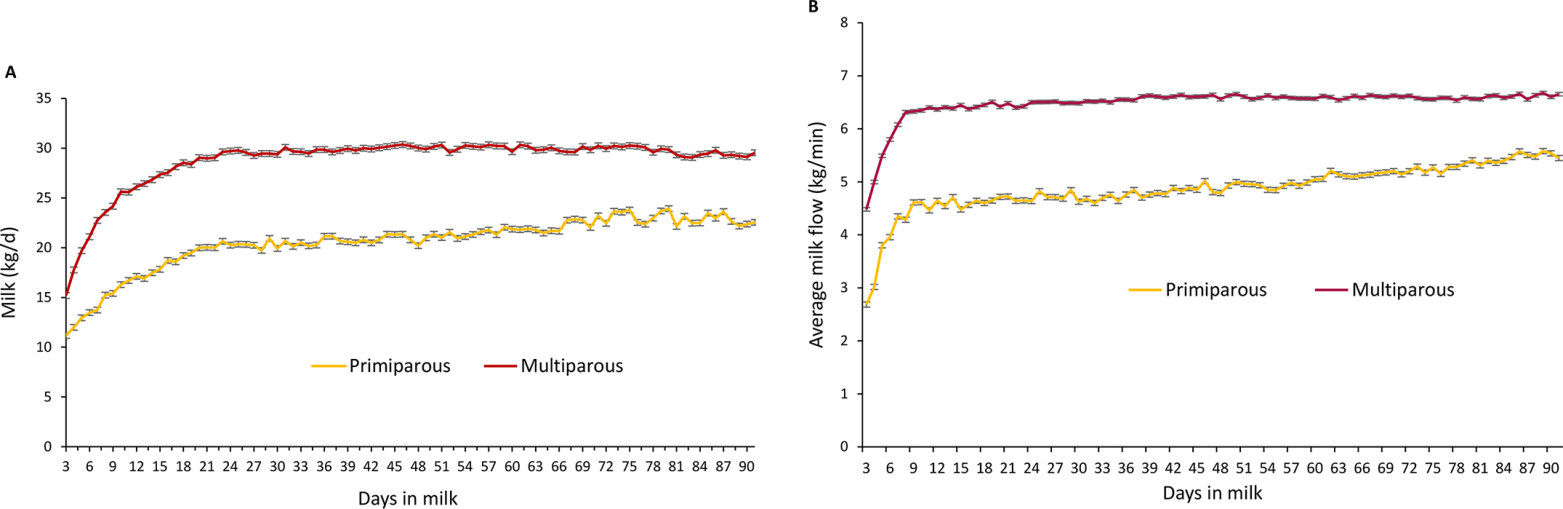
Daily milk yield (kg/d; A) and average milk flow (kg/min; B) of primiparous and multiparous cows during the first 90 DIM. Bars represent SE.

Average (SE) number of days with a KO event were 11.0 (0.77) d and 4.5 (0.36) d for PRI and MUL cows, respectively (*P* < 0.001). Contrary to the findings reported by O'Callaghan (1996), no differences in milk yield were established among number of days with KO categories. Average milk yields per day by quartile category of KO in PRI cows were 30.6 kg (Q1), 27.4 kg (Q2), 29.5 kg (Q3), and 28.2 kg (Q4) (*P* = 0.31), whereas average milk yields in MUL cows were 38.9 kg (Q1), 40.1 kg (Q2), 39.8 kg (Q3), and 38.3 kg (Q4) (*P* = 0.22).

Figure 2 shows the daily proportions of PRI and MUL cows experiencing KO events during the study period. For the overall monitoring period, the odds of KO were greater for PRI versus MUL cows (OR [95% CI] = 2.07 [1.58–2.73], *P* < 0.0001). In addition, Table 1 presents the adjusted OR and 95% CI for KO in PRI versus MUL cows by weekly periods. When KO was analyzed by DIM, proportions of cows kicking were greater in PRI than in MUL during the whole monitoring period. In PRI, proportions of KO increased from 0.10/d to 0.20/d between 3 DIM and 15 DIM, before beginning to decrease around 30 DIM and remaining above MUL up to 90 DIM. On the contrary, in MUL cows, proportions of KO remained close to 0.05/d during the 90-d period (Figure 2).

**Figure 2:**
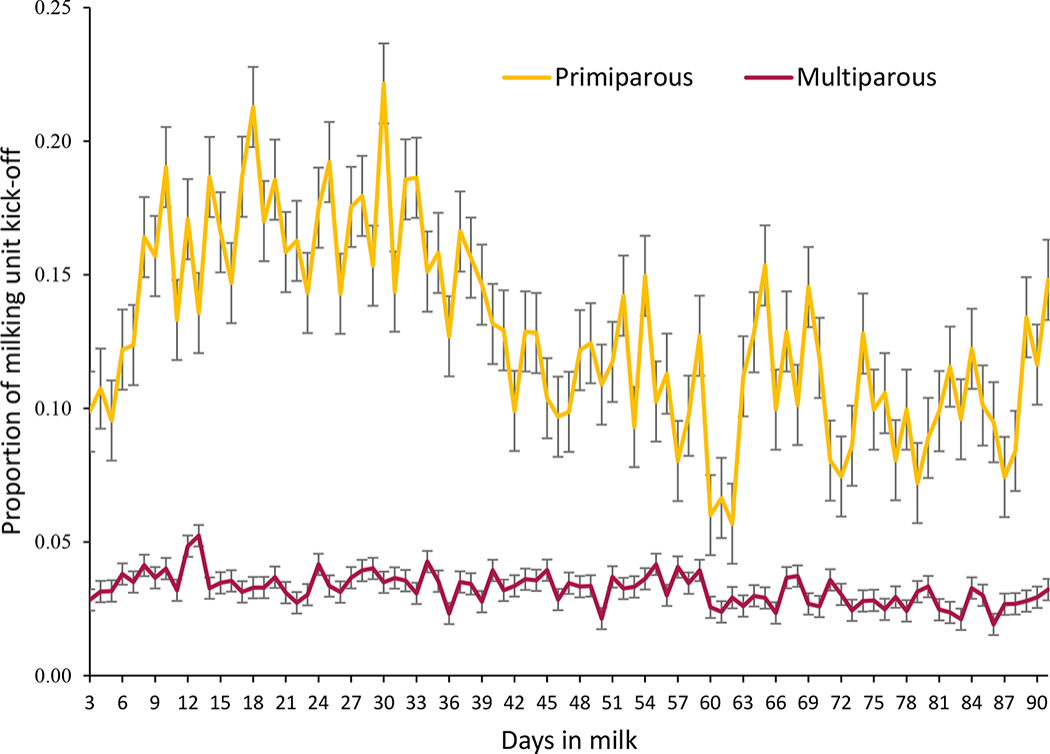
Proportions (SE) of milking unit kick-off events during the first 90 DIM, separated by primiparous and multiparous cows. Milking kick-off data were analyzed by grouping the 3 consecutive milking sessions in each day and categorized as yes or no, indicating whether or not an individual kicked at least once in a given day. The statistical models included parity (primiparous; multiparous), season of calving, and calving difficulty as fixed effects. Interactions tested were not significant and were removed from the models.

**Table 1:** Adjusted odds ratios (OR) and 95% CI for milking unit kick-off in primiparous versus multiparous (reference) cows by period of time^1^

Time period (DIM)	OR	95% CI	*P*-value
Overall (3–90)	2.07	1.58–2.73	<0.0001
3–7	1.57	1.07–1.22	0.0202
8–14	2.05	1.50–2.81	<0.0001
15–21	2.74	2.00–3.74	<0.0001
22–28	2.62	1.88–3.65	<0.0001
29–35	2.48	1.81–3.41	<0.0001
36–42	2.22	1.54–3.19	<0.0001
43–49	1.79	1.23–2.59	0.0023
50–56	1.86	1.27–2.74	0.006
57–63	1.41	0.94–2.13	0.1
64–70	2.53	1.73–3.71	<0.0001
71–77	1.71	1.11–2.62	0.014
78–84	1.98	1.29–3.03	0.0016
85–90	2.09	1.42–3.08	0.0002

1 Models were fitted for each of the time periods in analysis. The statistical models included parity (primiparous; multiparous), season of calving, and calving difficulty as fixed effects. Interactions tested were not significant and were removed from the models.

As hypothesized in this study, milking unit KO was greater in PRI than in MUL during the whole monitoring period. This agrees with findings reported by Cerqueira et al. (2017), where the odds of kicking were more than double in PRI compared with MUL cows. This was an expected result, considering that PRI cows had not been exposed to the milking routine previously and needed to habituate to the process.

Some research suggests that both extremely fearful and extremely calm cows are less reactive during milking than those in between (Bremner, 1997; Sutherland and Dowling, 2014). This is a possible explanation for the trends found in the present study, with inexperienced PRI cows (who are particularly stressed) displaying similar rates of KO compared with experienced MUL cows (who are often particularly calm) during the first days of lactation. Interestingly, occurrence of KO in PRI increased dramatically after the second week of lactation, whereas in MUL it remained close to 0.05/d throughout the 90-d monitoring period.

Anecdotally, it is often assumed that PRI cows will display dangerous behaviors most often during the first week of lactation before steadily decreasing, but the data from this study indicate that this may not be the case. This finding contradicts Bremner (1997), which found that PRI cows move and kick more frequently during the first 7 milkings than during subsequent milkings (Bremner, 1997). It is possible that these differences in cow behavior were caused by outside factors, such as milking equipment and parlor style, but more research is needed to clarify why cows in the present study showed increments in KO frequency during the first week of milking. By expanding the information given to workers regarding dairy cow behavior during early lactation, more realistic expectations would be formed and improve the welfare of both workers and PRI dairy cows during the milking routine.

Our findings are relevant for worker and cow welfare. With an increase in stress-related behaviors in PRI heifers during the transition period, workers often experience greater difficulty performing milking tasks and a higher risk for injury (Sorge et al., 2014; Edwards and Kuhn-Sherlock, 2021; Phillips et al., 2021). It has been shown that both the behavior and attitude of the handler can have a significant impact on the physiological stress indicators and behavior in stressful situations (Hemsworth et al., 2000; Waiblinger et al., 2003), and inaccurate expectations of PRI cow behavior has the potential to cause frustration, which can in turn further affect cow stress. In addition to implications for worker welfare, increased frequency of milking unit kick-off can affect production and cow health. Each time a milking unit is removed, there is a drop in vacuum and the cup must be reattached by a milking employee, interrupting the routine and decreasing efficiency of milking (Wieland et al., 2020). Additionally, it has been shown that milking unit kick-off can increase cross contamination and risk of intramammary infection (Baxter et al., 1992; Thompson, 2019), which in turn can affect both cow comfort and milk production (Leslie et al., 2010; Rollin et al., 2015; Suárez et al., 2017). In this study, mastitis presentation was partially associated with KO categories. The percentages of cows with at least one clinical mastitis case within 90 DIM by category of KO were 18.7% (Q1), 20.0% (Q2), 4.50% (Q3), and 24.4% (Q4) (*P* = 0.10) in PRI cows, whereas in MUL the percentages of affected cows were 9.56% (Q1), 7.45% (Q2), 8.75% (Q3), and 23.5% (Q4) (*P* < 0.0001). In agreement with previous research (Baxter et al., 1992; Thompson, 2019), in both parity groups, the percentage of cows affected with mastitis was greater in cows grouped in the quartile with more frequent KO events (Q4).

It is interesting to note that some research indicates a correlation between increased KO and high milk yield (O'Callaghan, 1996). However, despite MUL cows typically producing a higher yield than PRI, the research from the present study indicates that PRI cows experienced more than twice as much KO than MUL during the whole monitoring period. Moreover, no differences in milk yield were established among KO categories within parity.

With implications for worker welfare, animal welfare, and productivity in the milking parlor, an increased understanding of the relationship between KO and parity is important to the industry. Most previous studies focused on cow behavior at the milking parlor are based on visual observation (Rousing et al., 2004; Cerqueira et al., 2017). However, the advent of precision technologies creates opportunities for the monitoring of specific behaviors in large numbers of animals. In addition to the approach suggested in this study, kicking behavior during the milking procedure could be analyzed by use of leg-attached sensors or by the application of algorithms linked to video recording.

Although it is plausible to consider KO as an indication of adaptation to the milking routine, the lack of research supporting the assumption of KO as a proxy for habituation to the milking procedure is a limitation of the current study. Moreover, to the authors' knowledge, the level of correspondence between KO and actual kicking during the milking has not been investigated. In addition, there is possibility for variation for KO identification, depending on the definitions used by different manufacturers. In addition, due to the restrictions associated with the COVID-19 pandemic, the authors were unable to assess the presence udder edema, which is highly prevalent in PRI cows and may result in pain and discomfort, increasing KO behaviors. Other potentially relevant variables that were not assessed include teat placement and conformation, which may also have an effect on the likelihood of liner slip and KO (Martin et al., 2018).

Overall, levels of KO were consistently greater in PRI compared with MUL cows. Moreover, the relationship between DIM and the proportion of cows displaying KO was not linear, but rather increased for the first several weeks before decreasing again. This novel information has implications for training caretakers on what behaviors to expect from PRI cows as they begin their first lactation. A better understanding of this behavior using sensor and image technologies, as well as research exploring strategies to reduce incidences of KO during early lactation, could result in improved transition of first parity cows into milking.
